# Combination of TAPSE/sPAP Ratio and Myocardial Work to Assess Prognosis in Patients with Pulmonary Arterial Hypertension

**DOI:** 10.3390/jcdd13070324

**Published:** 2026-07-10

**Authors:** Jian Wang, Yingying Xu, Zhenwei Li, Hanbin Cui

**Affiliations:** 1 Department of Cardiology, The First Affiliated Hospital of Ningbo University, Ningbo 315000, China; wangjian1995@sjtu.edu.cn (J.W.); 15168117052@163.com (Z.L.); 2 Department of Cardiology, Shanghai Jiao Tong University School of Medicine Affiliated Renji Hospital, Shanghai 200127, China; 3 Department of Intensive Care Unit, The First Affiliated Hospital of Ningbo University, Ningbo 315000, China; 13857838664@163.com; 4 Ningbo Clinical Research Center for Cardiovascular Diseases, Ningbo 315000, China

**Keywords:** pulmonary arterial hypertension, TAPSE/sPAP ratio, right ventricular myocardial work, prognosis, echocardiography

## Abstract

Objective: Pulmonary arterial hypertension (PAH) is a progressive disease leading to right ventricular (RV) hypertrophy and failure. This study aims to evaluate the prognostic value of combining the tricuspid annular plane systolic excursion/systolic pulmonary artery pressure (TAPSE/sPAP) ratio with right ventricular myocardial work (RVMW) parameters in patients with PAH, to improve early risk stratification. Methods: A total of 43 PAH patients diagnosed via right heart catheterization were enrolled. Echocardiography-derived TAPSE/sPAP ratio and RVMW parameters, including right ventricular global work efficiency (RVGWE), global work index (RVGWI), global constructive work (RVGCW), and global wasted work (RVGWW), were measured. Clinical worsening events were recorded during a median 515-day follow-up. Statistical analyses included correlation tests, Firth penalized logistic regression, receiver operating characteristic (ROC) curves, and Kaplan–Meier survival analysis. Results: The TAPSE/sPAP ratio correlated negatively with RVGCW (r = −0.346, *p* = 0.023) and RVGWW (r = −0.417, *p* = 0.005), but not with RVGWE or RVGWI. Clinical worsening events occurred in 25.6% of patients, with significantly lower TAPSE/sPAP ratio (0.16 vs. 0.24 mm/mmHg), RVGWE (72.0% vs. 88.5%), and RVGWI (431.0 vs. 641.0 mmHg%) in the Event group (all *p* < 0.05). Multivariate Firth penalized logistic regression was used for combining TAPSE/sPAP ratio with RVGWE. ROC analysis demonstrated that the combination of TAPSE/sPAP and RVGWE yielded superior predictive power (AUC = 0.949, *p* < 0.001) compared to individual parameters. Conclusion: Non-invasive assessment of TAPSE/sPAP ratio and RVGWE provides significant prognostic value in PAH. Their combination enhances early risk prediction, offering a practical tool for clinical management.

## 1. Introduction

Pulmonary arterial hypertension (PAH) is a chronic and progressive disease, which is characterized by pulmonary vascular proliferation and continuous increases in resistance [1]. Sustained elevated afterload during disease progression leads to right ventricular (RV) hypertrophy and, ultimately, right heart failure (RHF) [2]. A study of hospital admissions for PAH patients between 2000 and 2009 reveals that the primary cause of hospitalization is RHF (56%), with a mortality rate of 13%, 26%, and 35% at 3rd, 6th, and 12th months following discharge, respectively [3]. Assessment of RV function is critical for prognosis in patients with PAH.

RV–pulmonary arterial (PA) coupling is an emerging concept. There is a growing recognition among scholars of the necessity to assess RV function by considering the RV and pulmonary circulation as a whole [4,5]. The ratio of RV end-systolic elastance (Ees) and PA elastance (Ea), as determined by invasive pressure–volume loop, serves as the gold standard for the evaluation of RV-PV coupling [6]. However, the limitations imposed on clinical practice by disadvantages such as invasion and time consumed in pressure–volume curves are a matter of significant concern. Recently, studies have shown that the TAPSE/sPAP ratio from echocardiography can effectively substitute the Ees/Ea ratio [7,8].

Myocardial work (MW) is another parameter, first proposed by Urheim et al. [9] to quantify ventricular systolic function. Subsequently, as improved by Russell et al. [10], a non-invasive pressure–strain loop (PSL) can effectively quantify MW. Richter et al. [11] have demonstrated that the echocardiographic pressure–strain loop-derived stroke work of RV correlates with the gold-standard invasive RV pressure–volume loop-based assessment of stroke work. RVMW relying on, independent of afterload, is superior to other conventional echocardiography parameters.

The TAPSE/sPAP ratio provides a load-adjusted estimate of RV function, reflecting the coupling between the RV and pulmonary circulation. However, it is primarily influenced by afterload and may not fully capture intrinsic myocardial contractile efficiency. In contrast, right ventricular global work efficiency (RVGWE), derived from non-invasive pressure–strain loops, quantifies the proportion of constructive work and is relatively less dependent on loading conditions. Therefore, we hypothesized that combining TAPSE/sPAP (load-dependent) with RVGWE (load-independent) would provide complementary insights into RV’s adaptation to afterload, enabling a more accurate identification of early RV decompensation. The primary objective of this study is to evaluate whether this combined echocardiographic model improves early risk stratification for clinical worsening events in patients with PAH, compared to the use of either parameter alone.

## 2. Methods

### 2.1. Study Population

A total of 43 patients diagnosed with PAH via right heart catheterization and treated at Shanghai Renji Hospital from January 2019 to June 2020 were enrolled in this study. The following diagnostic criteria for right heart catheterization (RHC) were stipulated in the 2022 ESC/ERS Guidelines of PAH [1]: mean pulmonary arterial pressure (mPAP) > 20 mmHg, pulmonary artery wedge pressure (PAWP) ≤ 15 mmHg and pulmonary vascular resistance (PVR) > 2 Wood. Other data from right heart catheterization, including pulmonary artery systolic pressure (PASP), pulmonary artery diastolic pressure (PADP), cardiac output (CO), and cardiac index (CI), as well as basic clinical information such as gender, age, etiology classification, and specific drug therapy were obtained from the electronic medical record system. Within one week prior to the echocardiography examination, brain natriuretic peptide (BNP) levels, 6 min walking distance (6MWD), and World Health Organization functional class (WHO-FC) were determined by a clinical investigator. The four-strata risk stratification for PAH patients was determined in accordance with the latest guideline protocol [1,12]. In summary, the following scale was used to assign points: 6MWD > 440, 320–440, 165–319, and <165 m were scored as 1, 2, 3, and 4 points, respectively. Similarly, the WHO-FC I, II, III, and IV were scored as 1, 1, 3, and 4 points, respectively. Finally, the BNP levels were scored as 1, 2, 3, and 4 points, with values of <50, 50–199, 200–800, and >800 ng/L, respectively. It is imperative to note that at least two of the aforementioned three variables must be incorporated into the risk stratification process. The rounded average value is designated as the risk point. The final calculated risk points for 1, 2, 3, and 4 were, respectively, low, intermediate–low, intermediate–high, and high. This study involving human participants was reviewed and approved by the Clinical Ethics Committee of Shanghai Renji Hospital (No.SK2020-55). The patients provided their written informed consent to participate in this study. The study was conducted in full accordance with the Declaration of Helsinki.

### 2.2. Right Heart Echocardiography

Each participant underwent standardized transthoracic echocardiography by a Vivid E95 ultrasound machine (GE Healthcare, Horten, Norway) equipped with an M5S probe. According to the American Society of Echocardiography [13], the tricuspid regurgitation velocity was measured in the right ventricle-focused apical four-chamber view, and sPAP was calculated using the simplified Bernoulli equation. TAPSE was determined by measuring the displacement of the tricuspid annulus during systole in M-mode. Furthermore, the TAPSE/sPAP ratio was calculated through the above two parameters. The right ventricular end-diastolic area and the right ventricular end-systolic area were delineated separately, and FAC was subsequently calculated. RAA was obtained at end-systole. Right ventricular and left ventricular basal diameter were measured at end-diastole in the four-chamber view to calculate RV/LV.

### 2.3. Right Ventricular Myocardial Work Analysis

The quantitative analysis of RVMW was performed using the offline workstation (EchoPAC version 203, GE Healthcare, Horten, Norway), as Butcher et al. previously described [14]. The timings of opening and closure of the pulmonic valve were identified by pulsed-wave Doppler in the parasternal short-axis view, while the timings of open and closure of the tricuspid valve were identified visually in the right ventricle-focused apical four-chamber view. Endocardial borders were automatically traced in the right ventricle-focused apical four-chamber view and were manually adjusted to achieve optimal results. The right ventricular global longitudinal strain (RVGLS) was assessed using two-dimensional speckle tracking analysis. In the myocardial work mode, the peak right ventricular pressure was designated as sPAP. The software subsequently outputs right ventricular global work index (RVGWI), right ventricular global constructive work (RVGCW), right ventricular global wasted work (RVGWW), and RVGWE. RVGWI represents the total right ventricular myocardial work during the interval, from tricuspid valve closure to tricuspid valve opening. RVGCW was defined as the work accomplished by myocardial shortening during systole in addition to the work achieved by myocardial lengthening during isovolumic relaxation. Conversely, RVGWW referred to the work executed by myocardial lengthening during systole, in addition to the work carried out by myocardial shortening during isovolumic relaxation. The efficiency of the right ventricle can be expressed as RVGWE, which was calculated as RVGCW divided by the sum of RVGCW and RVGWW.

### 2.4. Follow-Up

Patients were followed up every three months through telephone consultations or outpatient visits until May 2021. Clinical worsening events were defined as all-cause of mortality, hospitalization due to PAH, and the addition of new targeted drugs [15,16].

### 2.5. Statistical Analysis

Kolmogorov–Smirnov test was employed to test the normality of distribution. Normally distributed continuous variables were presented as mean ± standard deviation, while non-normally distributed continuous variables were presented as median (first and third quartiles). Categorical variables were expressed as numbers (percentage). Spearmen correlation coefficients were tested to analyze the relationship between TAPSE/sPAP ratio with RVMW parameters. At the end of follow-up, Student’s t-test was used to compare normally distributed continuous variables, while Mann–Whitney U test was used to compare non-normally distributed continuous variables between Non-event group and Event group. Univariate Firth penalized logistic model regression and least absolute shrinkage and selection operator (LASSO) regression analysis were conducted to analyze risk factors of adverse outcome in patients. Considering the limited sample size (*n* = 43) and the relatively small number of clinical worsening events (*n* = 11), which may lead to small-sample bias and potential complete separation in standard maximum likelihood estimation, multivariate Firth penalized logistic regression was used for combining TAPSE/sPAP ratio with RVGWE [17,18]. The calibration of the model was evaluated by calibration curve and Hosmer–Lemeshow test. Then, receiver operating characteristic (ROC) curve was performed to acquire area under the curve (AUC). Cut-off values of risk factors were identified by the Youden index, which was calculated by sensitivity + specificity-1. Decision curve analysis (DCA) was used to evaluate the clinical effectiveness of the model. Survival curves were generated using Kaplan–Meier method and compared using log-rank test. Statistical analysis was performed with SPSS 21.0 (IBM Corp., Armonk, NY, USA) and R version 4.5.1 (R Foundation for Statistical Computing, Vienna, Austria). Figures were produced with GraphPad Prism 8 (GraphPad Software, La Jolla, CA, USA). For all tests, a 2-tailed *p*-value of <0.05 was considered to be statistically significant.

## 3. Results

### 3.1. Clinical Characteristic, TAPSE/sPAP Ratio, and Myocardial Work Parameters

Demographic, clinical, and echocardiographic characteristics are shown in Table 1. TAPSE/sPAP ratio was 0.21 (0.16, 0.35) mm/mmHg in patients. RVGWE was 85.0 (76.0, 93.0)%, RVGWI was 581.0 (373.0, 793.0) mmHg%, RVGCW was 798.0 (621.0, 1186.0) mmHg%, and RVGWW was 149.0 (60.0, 220.0) mmHg%.

Here, 6MWD, 6 min walking distance; BNP, brain natriuretic peptide; FAC, fractional area change; PAH, pulmonary arterial hypertension; RAA, right atrial area; RV/LV, right ventricle/left ventricle basal diameter ratio; RHC, right heart catheterization; RVGCW, right ventricular global constructive work; RVGLS, right ventricular global longitudinal strain; RVGWE, right ventricular global work efficiency; RVGWI, right ventricular global work index; RVGWW, right ventricular global wasted work; sPAP, systolic pulmonary artery pressure; TAPSE, tricuspid annular plane systolic excursion; WHO-FC, World Health Organization functional class.

### 3.2. Relationship of TAPSE/sPAP Ratio and Myocardial Work Parameters

**Figure 1:**
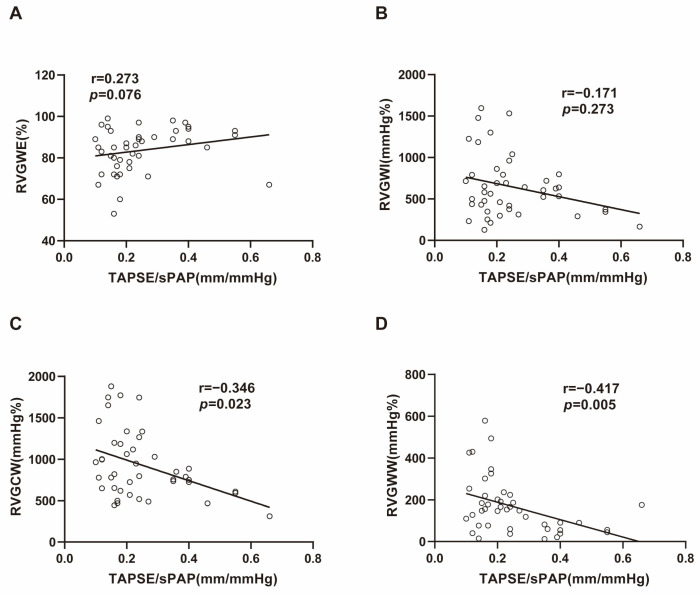
Correlation between TAPSE/sPAP ratio and myocardial work parameters. Spearmen correlation coefficients were tested to analyze the relationship between TAPSE/sPAP ratio with RVGWE (**A**), RVGWI (**B**), RVGCW (**C**), and RVGWW (**D**). All abbreviations as in Table 1.

Linear correlation analysis was used to assess the relationship between TAPSE/sPAP ratio and RVMW parameters in PAH patients, which are presented in Figure 1. TAPSE/sPAP ratio presented a negative correlation with RVGCW (r = −0.346, *p* = 0.023) and RVGWW (r = −0.417, *p* = 0.005). However, there was no significant correlation between TAPSE/sPAP ratio and RVGWE (r = 0.273, *p* = 0.076) or RVGWI (r = −0.171, *p* = 0.273).

### 3.3. The Value of TAPSE/sPAP Ratio and Myocardial Work Parameters in Adverse Outcome Prediction

**Figure 2:**
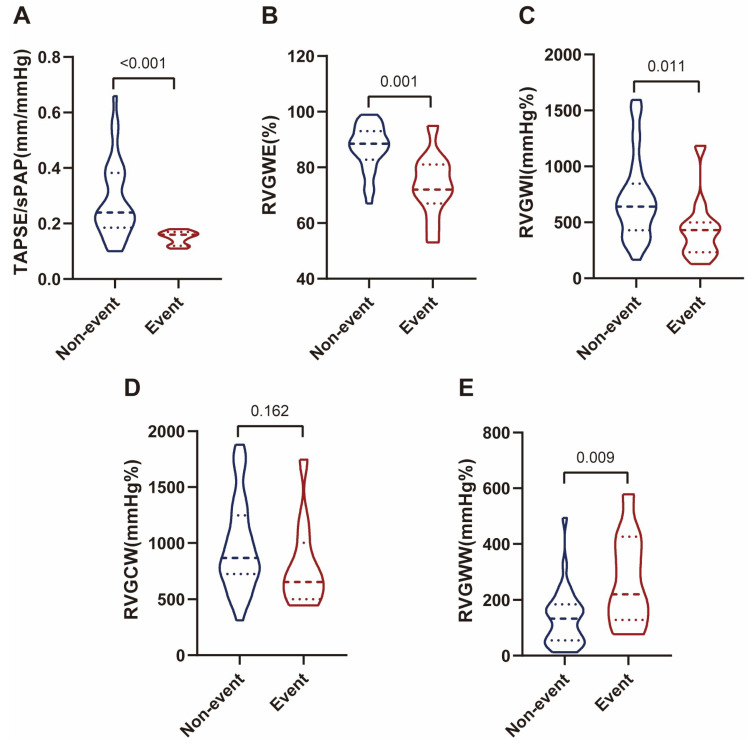
Difference in TAPSE/sPAP ratio and myocardial work parameters between Non-event group and Event group. Mann–Whitney U test was used to compare TAPSE/sPAP ratio (**A**), RVGWE (**B**), RVGWI (**C**), RVGCW (**D**), and RVGWW (**E**). All abbreviations as in Table 1.

In the course of a 515-day (385, 535) follow-up period, clinical worsening events occurred in 11 patients (25.6%): 9 patients (20.9%) had hospitalization due to PAH, and 2 patients (4.6%) had an addition of new targeted drugs. The 43 patients were categorized into two groups: Non-event group (*n* = 32) and Event group (*n* = 11). As shown in Figure 2, TAPSE/sPAP ratio [0.24 (0.19, 0.38) vs. 0.16 (0.12, 0.17) mm/mmHg; *p* < 0.001], RVGWE [88.5 (82.8, 93.0) vs. 72.0 (67.0, 81.0)%; *p* = 0.001], and RVGWI [641.0 (429.3, 845.8) vs. 431.0 (233.0, 499.0) mmHg%; *p* = 0.011] were significantly decreased in the Event group compared with Non-event group, while RVGWW [133.0 (55.3, 184.3) vs. 220.0 (128.0, 426.0) mmHg%; *p* = 0.009] significantly increased. There was no significant difference but a decreased trend between the two groups in RVGCW [868.5 (726.0, 1248.0) vs. 654.0 (500.0, 1002.0) mmHg%; *p* = 0.162].

**Figure 3:**
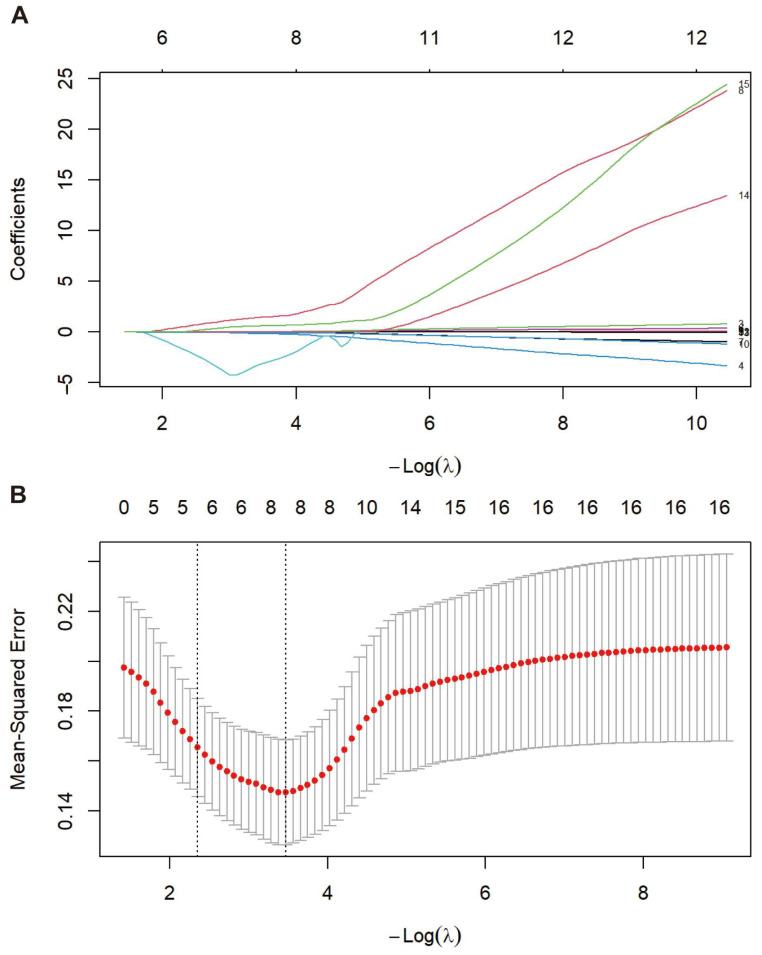
Selecting clinical features based on the least absolute shrinkage and selection operator (LASSO) logistic regression. (**A**) Profiles of LASSO coefficients for the 15 features. This plot shows the coefficient profile against the logarithmic (lambda) sequence. (**B**) The optimal parameter (lambda) is selected using 10-fold cross-validation with minimum criteria in the LASSO logistic regression. Each red dot represents a lambda (λ) value on the path, and confidence intervals for the error rate are indicated. In drawing the black vertical lines, we used the minimum criteria and the one standard error of the minimum criteria (1-SE) to determine the optimal values.

Based on the results of univariable Firth penalized logistic analyses (Table 2) and clinical significance, the LASSO regression was performed on 15 potential variables. Using the linear combination of factors weighted by coefficients assigned to each subject, a risk score was calculated for each subject, from which a coefficient distribution curve was derived (Figure 3A). The cross-validation error graph for the LASSO regression model can be viewed in Figure 3B. Then, we identified seven predictive variables, including BNP, WHO-FC III, TAPSE, TAPSE/sPAP ratio, RV/LV, RVGLS, and RVGWE. Finally, to avoid overfitting given the limited sample size, and by utilizing multivariable Firth penalized logistic regression, a model incorporating TAPSE/sPAP ratio and RVGWE was developed (Table 2).

### 3.4. Combination of TAPSE/sPAP Ratio and Myocardial Work Parameters in Adverse Outcome Prediction

**Figure 4:**
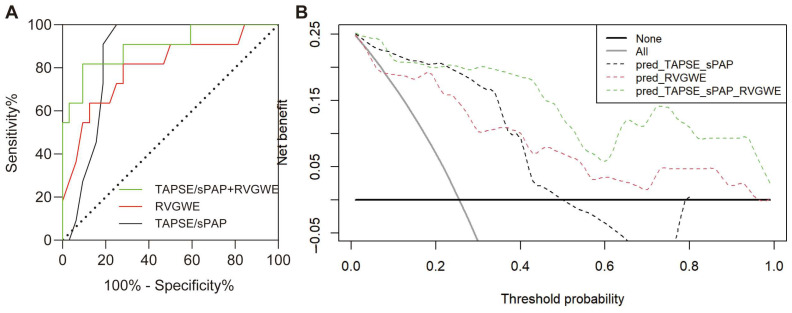
ROC analysis and decision curve analysis of patients with PAH. (**A**) ROC analysis of patients with PAH. (**B**) Decision curve analysis of patients with PAH.

**Figure 5:**
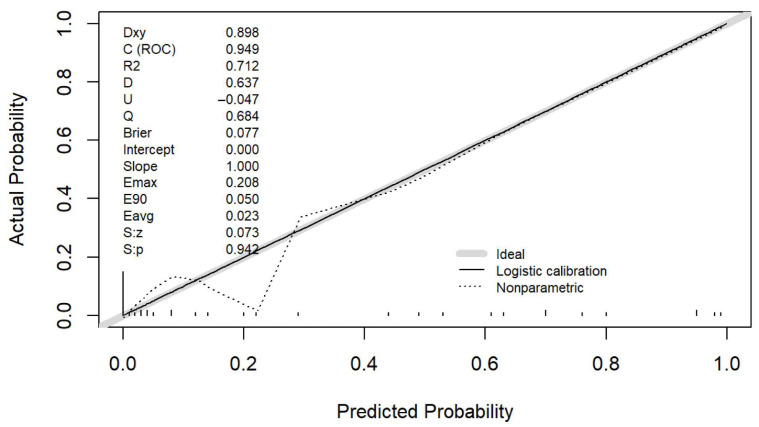
Calibration curves for the model.

ROC analysis demonstrated that the combination of TAPSE/sPAP ratio with RVGWE yielded higher predictive adverse outcome values than the single variables before combination (AUC = 0.949, *p* < 0.001), as depicted in Table 3 and Figure 4A. The results of the DCA on a visual basis confirmed that the model had superior overall net benefits within a wide, practical threshold probability range (Figure 4B). In addition, observations and predictions of adverse outcome correlated well with the calibration plots (Figure 5, Hosmer–Lemeshow R^2^ = 1.92, *p* = 0.992).

**Figure 6:**
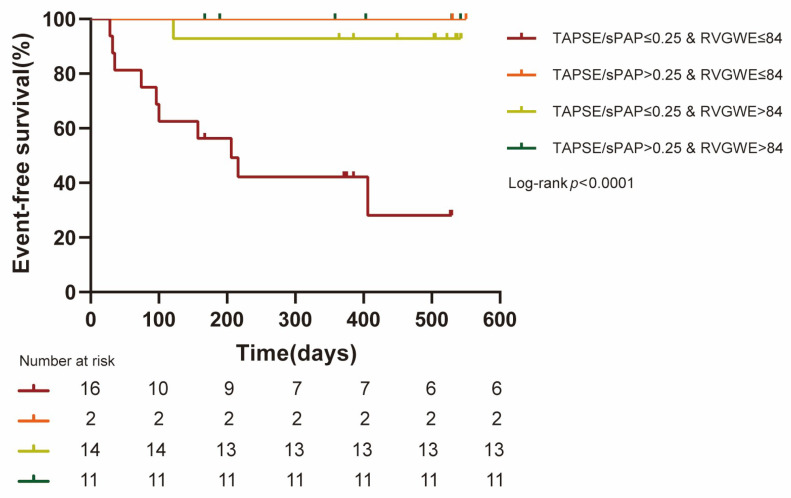
Kaplan–Meier survival analysis of patients with PAH.

The optimal critical values of TAPSE/sPAP ratio and RVGWE were obtained by the maximum Youden index as 0.25mm/mmHg and 84.0%, respectively. Kaplan–Meier survival analysis showed that patients with TAPSE/sPAP ≤ 0.25mm/mmHg and RVGWE ≤ 84.0% (log-rank *p* < 0.0001) were more susceptible to adverse outcomes (Figure 6).

## 4. Discussion

Typically, RV is coupled to the low-resistant and highly dilated pulmonary vessel [19]. In contrast to the left ventricle (LV), RV has a weaker wall and is more compliant [20,21]. Therefore, the RV is more likely to tolerate volume load but not pressure load. In the initial phases of PAH which exerted chronic pressure on RV, RV-PA coupling may be diminished, despite the preservation of normal RV function and the potential augmentation of contractility. This phenomenon can be justified in our present study. In the correlation analysis, a decrease in the TAPSE/sPAP ratio is accompanied by an increase in both RVGCW and RVGWW, with the former displaying a stronger correlation with RVGWW compared to RVGCW. This observation suggests a potential progression from cardiac compensation to dysfunction as RV-PA uncoupling occurred.

Complex pathophysiological processes, including myocyte hypertrophy, fibrosis, ischemia, neurohormonal activation, inflammation, and metabolic substrate shifts, are involved in the progression from RV dysfunction to RHF [22]. Initially, RV hypertrophy is adaptive and accompanied by an increase in contractility and preserved stroke volume [23]. Eventually, fibroblasts replace cardiomyocytes, oxygen demand exceeded supply, and contractility is further reduced, leading to complete RV-PA uncoupling [24]. In our present study, TAPSE/sPAP ratio, RVGWE, and RVGWI are significantly lower in the Event group than Non-event group, whereas RVGWW was higher. Interestingly, there is no significant difference in RVGCW between the Event and Non-event groups. Li et al. [25] also reported that there was no significant difference in RVGCW between PAH patients at low risk and intermediate–high risk. This phenomenon further suggests that RVGCW cannot increase further during the progression from RV dysfunction to RHF. In contrast, once RVGCW increases dramatically, clinical worsening events occur.

The 2022 ESC/ERS Guidelines recommend TAPSE/sPAP ratio for the assessment of RV function in patients with PAH [1]. Two studies have demonstrated a direct correlation between TAPSE/sPAP ratio and prognosis in patients with PAH [26,27]. To our knowledge, RVMW has not been extensively studied in PAH. The phenomenon of impaired RVMW associated with adverse prognosis in patients with pulmonary hypertension was confirmed by Butcher et al. [28] In our multivariate Firth penalized logistic regression analysis, TAPSE/sPAP ratio and RVGWE are independent predictors of prognosis in patients with PAH. Furthermore, the ROC curves indicate that values of 0.25 mm/mmHg and 84.0 mmHg% can be used as critical predictors of the transition from cardiac compensated to decompensated phases for TAPSE/sPAP ratio and RVGWE, respectively.

TAPSE/sPAP ratio and RVMW can be non-invasive and convenient assessments of RV function, as described above. However, the role of combined evaluation in clinical practice has not been elucidated. In fact, a combination of multiple parameters proved to be superior to assessment of independent factors. Ghio et al. [29] proposed that the integration of TAPSE, tricuspid regurgitation grade, and inferior vena cava derived from echocardiography provided good stratification of all-cause mortality in PAH patients. Furthermore, this comprehensive risk stratification model demonstrated superiority over the single echocardiographic parameters recommended by current guidelines for PAH. Li et al. [24] combined multiple conventional and novel echocardiographic parameters to stratify the risks of PAH patients. In the present study, it is noteworthy that the combination of TAPSE/sPAP ratio and RVGWE demonstrated a superior capacity for predicting the prognosis of patients with PAH when compared to these parameters utilized independently. Clinical management may benefit from this finding.

Several limitations of the present study should be acknowledged. First and foremost, this was a single-center study with a relatively small sample size (*n* = 43) and a limited number of clinical events (*n* = 11). This raises concerns regarding the reliability of the multivariate predictive model. Specifically, the events-per-variable (EPV) ratio was low, which increases the risk of overfitting and may have inflated the apparent predictive performance (e.g., the AUC of 0.949). To mitigate these estimation biases, we employed Firth penalized logistic regression; however, this statistical correction cannot fully compensate for the limited sample size. Therefore, the reported predictive performance should be interpreted cautiously as exploratory, and external validation in independent, larger cohorts is urgently required. Second, the population composition is predominantly concentrated in connective tissue disease associated with PAH, with data bias present. Third, correlation analysis shows that there is a trend of correlation between the TPASE/sPAP ratio and RVGWE/RVGWI with no statistical significance, and subsequent studies should expand the sample size to further improve the data results. Fourth, due to the retrospective nature of data collection, right atrial pressure and the pulmonary artery pulsatility index were not consistently available for all patients, precluding their inclusion in the analysis. Prospective studies with standardized hemodynamic assessments are warranted to further evaluate the relationship between these invasive parameters and non-invasive echocardiographic indices. Finally, this is a short-term prognostic study designed to provide preliminary joint reference for clinical purposes, with long-term prognostic studies to follow.

## 5. Conclusions

In conclusion, both the TAPSE/sPAP ratio and RVGWE derived from non-invasive echocardiography are significant independent predictors of clinical worsening in patients with PAH. Our findings demonstrate that their combination significantly enhances prognostic discrimination (AUC: 0.949) compared to the individual metrics alone. The identified optimal cut-off values of 0.25mm/mmHg for TAPSE/sPAP and 84.0% for RVGWE may serve as practical, accessible thresholds to help clinicians identify PAH patients at higher risk of early clinical deterioration, potentially guiding timely therapeutic intensification. However, given the exploratory nature of this single-center study with limited sample size, these promising findings require further validation through large-scale, prospective, multicenter studies before they can be incorporated into routine clinical risk stratification algorithms.

## Figures and Tables

**Table 1:** Clinical and echocardiographic characteristics in PAH patients.

Characteristic	PAH (*n* = 43)
Gender (% female)	41 (95.3)
Age (years)	41.6 ± 11.9
BNP (ng/L)	188.0 (41.0, 502.0)
6MWD (m)	433.1 ± 115.6
WHO-FC (%)	
I	13 (30.2)
II	13 (30.2)
III	14 (32.6)
IV	3 (7.0)
Etiology (%)	
Connective tissue disease associated with pulmonary arterial hypertension	33 (76.8)
Idiopathic pulmonary arterial hypertension	5 (11.6)
Congenital heart disease associated with pulmonary arterial hypertension	4 (9.3)
Portopulmonary hypertension	1 (2.3)
Specific drug therapy	
None (%)	2 (4.7)
Endothelin receptor antagonists (%)	33 (76.7)
Phosphodiesterase type 5 inhibitors (%)	33 (76.7)
Prostacyclin analogs (%)	5 (11.6)
Risk (%)	
Low risk	10 (23.3)
Intermediate–low risk	15 (34.9)
Intermediate–high risk	12 (27.9)
High risk	6 (13.9)
RHC data	
Pulmonary arterial systolic pressure (mmHg)	72.5 ± 19.1
Pulmonary arterial diastolic pressure (mmHg)	34.6 ± 8.1
Mean pulmonary arterial pressure (mmHg)	48.4 ± 10.6
Pulmonary artery wedge pressure (mmHg)	12.0 (8.0, 14.0)
Cardiac output (L/min)	4.6 ± 1.6
Cardiac index (L/min·m^2^)	2.9 ± 0.9
Pulmonary vascular resistance (dyn·s·cm^−5^)	651.2 (486.3, 1037.4)
Echocardiography measurements	
sPAP (mmHg)	73.9 ± 26.8
TAPSE (mm)	15.7 ± 3.6
TAPSE/sPAP (mm/mmHg)	0.21 (0.16, 0.35)
FAC (%)	31.5 ± 12.5
RAA (cm^2^)	16.2 (14.0, 20.1)
RV/LV	1.3 ± 0.4
RVGLS (%)	−13.7 ± 4.1
Myocardial work paraments	
RVGWE (%)	85.0 (76.0, 93.0)
RVGWI (mmHg%)	581.0 (373.0, 793.0)
RVGCW (mmHg%)	798.0 (621.0, 1186.0)
RVGWW (mmHg%)	149.0 (60.0, 220.0)

Values are mean ± SD, *n* (%), or median (first and third quartiles).

**Table 2:** Firth penalized logistic regression analysis of predictors associated with clinical worsening in PAH patients.

	Univariate Analysis			Multivariate Analysis		
Variable	OR	95% CI	*p*-Value	OR	95%CI	*p*-Value
Age (years)	0.998	0.940–1.055	0.951			
BNP (ng/L)	1.002	1.000–1.003	0.007			
6MWD (m)	0.994	0.988–1.000	0.067			
WHO-FC	/	/	/			
I	Ref.	Ref.	Ref.			
II	1.812	0.209–22.042	0.587			
III	8.333	1.404–91.092	0.018			
IV	5	0.311–87.152	0.238			
sPAP (mmHg)	1.025	0.999–1.056	0.052			
TAPSE (mm)	0.694	0.513–0.878	0.001			
TAPSE/sPAP (mm/mmHg)	<0.001	0.000–0.001	<0.001	<0.001	0.000–0.001	0.001
FAC (%)	0.904	0.823–0.970	0.003			
RAA (cm^2^)	1.091	0.994–1.241	0.066			
RV/LV	12.686	2.035–120.433	0.005			
RVGLS (%)	1.412	1.143–1.861	<0.001			
RVGWE (%)	0.879	0.793–0.949	<0.001	0.858	0.716–0.954	0.002
RVGWI (mmHg%)	0.997	0.994–1.000	0.028			
RVGCW (mmHg%)	0.999	0.997–1.001	0.257			
RVGWW (mmHg%)	1.007	1.002–1.014	0.006			

All abbreviations as in Table 1.

**Table 3:** Details of the receiver operator characteristic analysis to predict adverse outcome.

Parameter	AUC	*p*-Value	95% CI	Cut-Off Value	Sensitivity (%)	Specificity (%)
TAPSE/sPAP (mm/mmHg)	0.858	<0.001	0.747–0.969	0.25	100.0	75.0
RVGWE (%)	0.835	0.001	0.684–0.986	84.0	90.9	75.0
TAPSE/sPAP + RVGWE	0.949	<0.001	0.887–1.000	/	90.9	91.6

AUC, area under curve; other abbreviations as in Table 1.

## Data Availability

The original contributions presented in this study are included in the article material. Further inquiries can be directed to the corresponding author.
